# Green Synthesis of Silver Nanoparticles Using Extract of Oak Fruit Hull (Jaft): Synthesis and In Vitro Cytotoxic Effect on MCF-7 Cells

**DOI:** 10.1155/2015/846743

**Published:** 2015-01-01

**Authors:** Rouhollah Heydari, Marzieh Rashidipour

**Affiliations:** ^1^Razi Herbal Medicines Research Center, Lorestan University of Medical Sciences, 68149-89468 Khorramabad, Iran; ^2^Department of Chemistry, Faculty of Sciences, Islamic Azad University, Khorramabad Branch, Khorramabad 68149-68418, Iran

## Abstract

A green synthetic approach by using oak fruit hull (Jaft) extract for preparation of silver nanoparticles (AgNPs) was developed and optimized. Parameters affecting the synthesis of AgNPs, such as temperature, extract pH, and concentration of extract (ratio of plant sample to extraction solvent), were investigated and optimized. Optimum conditions for the synthesis of silver nanoparticles are as follows: Ag^+^ concentration, 1 mM; extract concentration, 40 g/L (4% w/v); pH = 9 and temperature, 45°C. Biosynthesized silver nanoparticles were characterized using UV-visible absorption spectroscopy (UV-Vis), Fourier-transform infrared spectroscopy (FT-IR), X-ray diffraction (XRD), dynamic light scattering (DLS), transmission electron microscopy (TEM), and scanning electron microscopy (SEM). TEM and DLS analyses have shown the synthesized AgNPs were predominantly spherical in shape with an average size of 40 nm. The cytotoxic activity of the synthesized AgNPs and Jaft extract containing AgNPs against human breast cancer cell (MCF-7) was investigated and the half maximal inhibitory concentrations (IC_50_) were found to be 50 and 0.04 *μ*g/mL at 24 h incubation, respectively. This eco-friendly and cost-effective synthesis method can be potentially used for large-scale production of silver nanoparticles.

## 1. Introduction

Green chemistry is the design of chemical products and processes that reduce or eliminate the use or generate hazardous substances for human health and environment. Therefore, green chemistry protects the environment, not by cleaning up, but by introducing new chemical processes that do not pollute the environment.

Nowadays, development of green synthesis of metallic nanoparticles and their applications is one of the most important areas of research. For synthesizing silver nanoparticles (AgNPs) several methods such as chemical synthesis [1], electrochemical synthesis [2], radiation synthesis [3, 4], photochemical synthesis [5], and biological synthesis [6–9] have been used. In comparison with other methods, biological methods for synthesis of AgNPs are environmental friendly. Due to this reason, these methods have been preferred. Among the biological methods, the use of plant extracts for the synthesis of AgNPs is not only simple and cost effective but also the synthesized particles are stable. Recently, a rapid, energy-efficient, green, and economically scalable room temperature method for synthesis of stable AgNPs by using the tannic acid (a polyphenolic compound derived from plant extract) was developed by Sivaraman et al. [10].

In several reports it was demonstrated that the antimicrobial activities of AgNPs are dependent on the size, shape, and stabilizing agents of nanoparticles. The antibacterial activities increased with size reduction of AgNPs [11, 12]. Aggregation of nanoparticles causes reduction in antibacterial activities of AgNPs. Therefore, combination of nanoparticles with stabilizer agents prevents the aggregation and leads to maintain antibacterial activities of AgNPs.

Despite the widespread use of AgNPs in medical fields, relatively few in vitro studies have been accomplished to determine the cytotoxicity effects of AgNPs on different cell lines including HeLa [13, 14], MCF-7 [15], NIH3T3 [16], and human cells [17]. In all these studies, AgNPs were removed from extract (plant extract) after the synthesis and their anticancer activities were studied. However, there is no study on anticancer activities of AgNPs in the presence of plant extracts. This is the first report on the anticancer activities of plant extracts (Jaft) containing AgNPs.

There are many reports on the therapeutic properties of fruit, leave, gall, and Jaft of oak due to presence of phenols, tannins, and proteins in this plant. Extract of* Quercus infectoria* was found to contain a large amount of polyphenols and possess a potent reducing power [18–22].

In this work, synthesis of AgNPs using aqueous Jaft extract of* Quercus infectoria* was investigated and optimized. The cytotoxicity effects of extract containing AgNPs on cell viability were studied by using human breast cancer cells (MCF-7).

## 2. Experimental

### 2.1. Chemicals

Silver nitrate, sodium hydroxide, ammonia solution (25%), and orthophosphoric acid were purchased from Merck Chemical Co. (Darmstadt, Germany). 2-(4,5-Dimethylthiazol-2-yl)-2,5-diphenyltetrazolium bromide (MTT) was purchased from Sigma-Aldrich (USA). All solutions were prepared with distilled water.

### 2.2. Instrumentation

UV-Vis spectra were recorded by using a UV-Vis spectrophotometer (Jenway, model 6505, UK). The existence of biomolecules in shell of synthesized AgNPs was investigated using FT-IR (BRUKER, model TENSOR 27, Germany) analysis. Biosynthesis of AgNPs was demonstrated by X-ray diffraction (X'Pert PRO, SciSpec Co. Thailand) analysis. The shape of the freeze dried AgNPs was analyzed by SEM and TEM (SEM, Hitachi model S-4160 and TEM, Philips model CM30). The particle size distribution and zeta potential analysis of biosynthesized AgNPs were evaluated via dynamic light scattering (DLS) and zeta potential analysis by using a Malvern Zetasizer Nano range instrument (Malvern Instruments Ltd., Malvern, UK).

### 2.3. Plant Sample and Preparation of Extract

Fruits of oak trees were collected from Khorramabad Mountains in the west of Iran. Oak fruit hull (Jaft) was isolated and dried in 25°C in shadow. The same sample was used in the whole optimization study.

In order for preparation of extract, 5.0 g of air-dried and pulverized Jaft was extracted by ultrasonic bath for 24 h with 50 mL distilled water and filtered by using Whatman filter paper. Finally, the filtrate was centrifuged for 10 min at 4000 rpm. The supernatant was used for the synthesis of AgNPs.

### 2.4. Biosynthesis of Silver Nanoparticles

40 mL (10% w/v) of Jaft aqueous extract, 10 mL of ammonia solution (1 M), and 10 mL of silver nitrate solution (10 mM) were mixed. The solution pH adjusts to the desired value by using sodium hydroxide or phosphoric acid solution and then was diluted until 100 mL with distilled water. The mixture was stirred for 4 h at 45°C.

### 2.5. Isolation of AgNPs from Extract

After centrifuging of AgNPs solution for 10 min at 10000 rpm, AgNPs were sedimented at the bottom of the conical tube. The supernatant phase was removed and AgNPs were washed with 10 mL water for three times. After the washing, the residue was transferred to freeze dryer. Finally, the obtained powder was subjected to XRD, FT-IR, SEM, and TEM analyses.

### 2.6. In Vitro Cytotoxicity of Synthesized AgNPs and Extract Containing AgNPs

Cell viability was calculated according to the method developed by Denizot and Lang [23] by using the reduction of MTT to formazan. MCF-7 and normal cells (human blood mononuclear cells) were treated with different concentration levels of biosynthesized AgNPs (dispersed in water) and dispersed AgNPs in Jaft extract. The treated cells were incubated for 24 h at 37°C for cell viability analysis. Finally, the treated cells were subjected to MTT assay. MTT stock concentration (5 mg/mL) was prepared in PBS, and 100 *μ*L of this solution was added to each AgNPs treated well and incubated for 4 h. Then, 100 *μ*L of dimethyl sulphoxide (DMSO) was added to each well, and the absorbance values were determined by spectrophotometer at 490 nm (Eliza MAT 2000, DRG Instruments, GmbH). Results were expressed as cell viability.

### 2.7. Data Analysis

All measurements were performed in triplicate. The one-way analysis of variance (ANOVA) was done for expressing experimental significance of results. In cell viability test the *P* values < 0.05 were considered as significant.

## 3. Results and Discussion

The main parameters affecting the formation of nanoparticles, including pH of extract, temperature of synthesis process, and extract concentration, were investigated and optimized.

### 3.1. Characterization of Biosynthesized AgNPs

**Figure 1:**
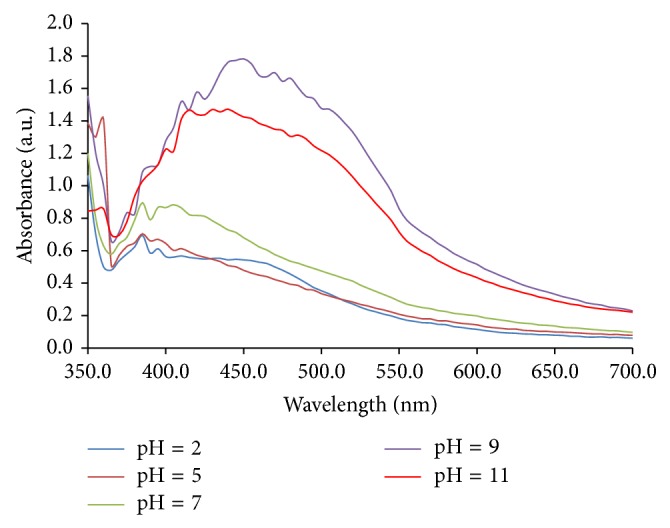
Effect of extract pH on AgNPs synthesis. Synthesis conditions: silver nitrate concentration, 1 mM; extract concentration; 40 g/L, temperature, 45°C.

In green synthesis of AgNPs using plant extracts, various constituents may contribute in reduction process of silver ions. Therefore, changing the chemical state (e.g., ionization) of these constituents can be affected on performance and rate of reduction process. For this reason, the effect of extract pH on the synthesis of AgNPs in the range of 2–11 was investigated by using UV-Vis spectrophotometer. The results (Figure 1) show that the rate of AgNPs synthesis increases with increasing pH up to pH = 9 and then decrease. The reason for this behavior may be due to the ionization of phenolic compounds and tannins in the extract of oak [18–22].

**Figure 2:**
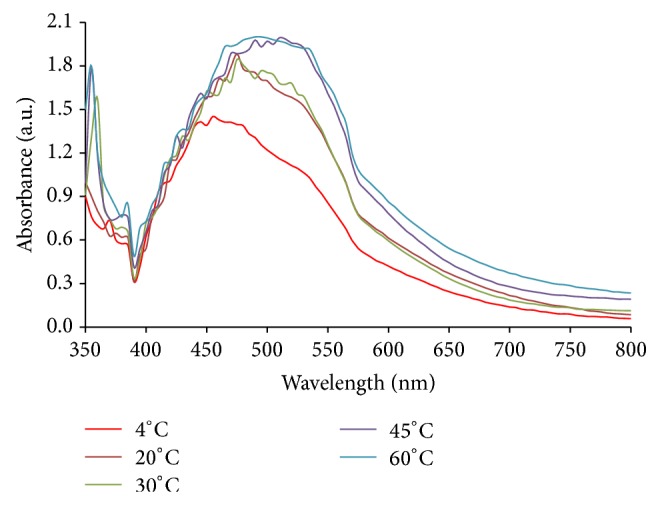
Effect of temperature on AgNPs synthesis. Synthesis conditions: silver nitrate concentration, 1 mM; extract concentration; 40 g/L, extract pH, 9.

Synthesis of silver nanoparticles was performed at different temperatures in the range of 4 to 60°C. The results in Figure 2 show that the efficiency of silver nanoparticles synthesis was highest at 45°C.

**Figure 3:**
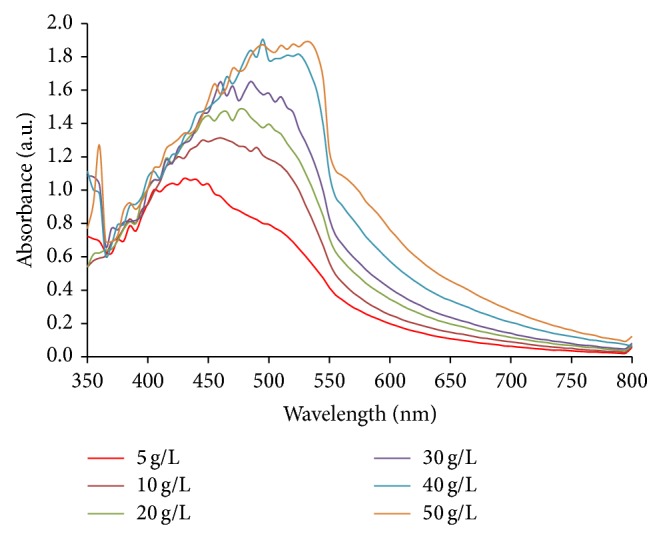
Effect of extract concentration on AgNPs synthesis. Synthesis conditions: silver nitrate concentration, 1 mM; temperature, 45°C, extract pH, 9.

In order to complete reduction of silver ions to silver nanoparticles, different concentrations of extract (Jaft extract) were mixed with a constant volume of silver nitrate (1 mM). The results in Figure 3 show that increasing the concentration of extract led to synthesizing more AgNPs and ultimately level off at concentration of 40 g/L.

**Figure 4:**
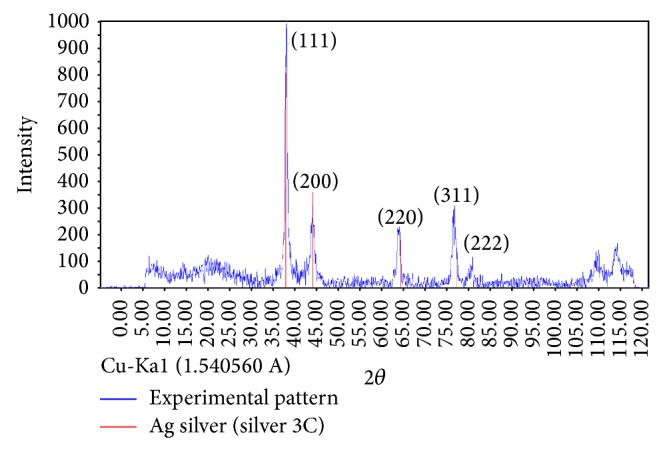
XRD pattern of biosynthesized AgNPs (blue) and reference XRD pattern of cubic silver (red). Synthesis conditions: silver nitrate concentration, 1 mM; temperature, 45°C, extract pH, 9; extract concentration, 40 g/L.

The XRD patterns of synthesized AgNPs are shown in Figure 4. In comparison with cubic Ag reference pattern, the spectra data reveal the five diffraction lines (111), (200), (220), (311), and (222), which are compatible with the standard pattern.

**Figure 5:**
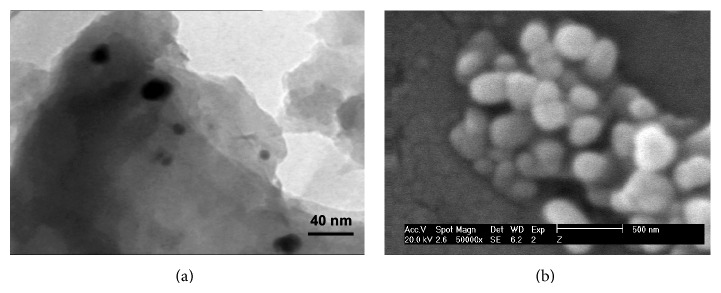
SEM (a) and TEM (b) images of biosynthesized AgNPs. Synthesis conditions: silver nitrate concentration, 1 mM; temperature, 45°C, extract pH, 9; extract concentration, 40 g/L.

**Figure 6:**
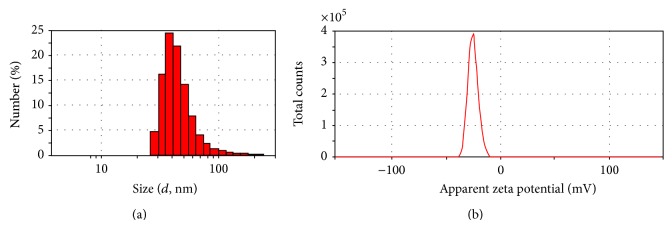
DLS analysis (a) and zeta potential (b) measurement of biosynthesized AgNPs. Synthesis conditions: silver nitrate concentration, 1 mM; temperature, 45°C, extract pH, 9; extract concentration, 40 g/L.

The SEM and TEM images show that the synthesized AgNPs are in spherical structures (Figure 5). The results of DLS analysis showed the average size of nanoparticles is 40 nm (Figure 6(a)). Zeta potential is an important parameter for understanding the state of the nanoparticle surface and predicting the long-term stability of the dispersion. According to previous reports, nanoparticles with zeta potential values greater than +25 mV or less than −25 mV typically have high degrees of stability. Dispersions with a low zeta potential value will eventually aggregate due to interparticle attractions. On the other hand, the zeta potential of nanoparticles strongly depends on pH and electrolyte concentration of the dispersion [13]. The zeta potential value of dispersed synthesized AgNPs in deionized water in absence of any electrolyte was −25.3 mV (Figure 6(b)).

**Figure 7:**
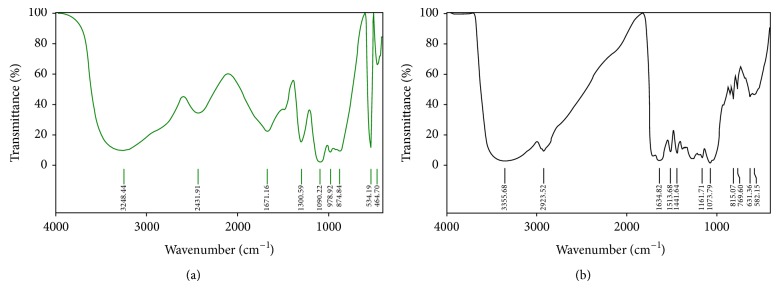
FT-IR spectra of Jaft extract (a) and biosynthesized AgNPs (b). Synthesis conditions: silver nitrate concentration, 1 mM; temperature, 45°C, extract pH, 9; extract concentration, 40 g/L.

Figure 7 shows the FT-IR spectra of dried Jaft aqueous extract and synthesized AgNPs. Presence of similar peaks with small shift in both spectra reveals the synthesized AgNPs are containing natural compounds from extract. Stability of AgNPs can be attributed to existence of these compounds in shell of nanoparticles.

### 3.2. In Vitro Cytotoxicity of Biosynthesized AgNPs and Extract Containing AgNPs

In previous reports [13, 15], the biosynthesized AgNPs were isolated from the extract and their anticancer effects were examined. In this study, the anticancer activities of biosynthesized AgNPs were compared with extract containing AgNPs.

**Figure 8:**
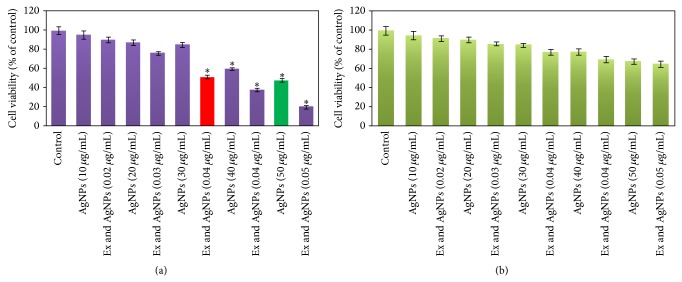
MTT assay results. (a) Cytotoxic effects of dispersed AgNPs in the Jaft extract and AgNPs on cancer cell line (MCF-7) and (b) cytotoxic effects of dispersed AgNPs in the Jaft extract and AgNPs on normal cell line (human blood mononuclear cells). Data are calculated as mean ± SD of three experiments. AgNPs: dispersed AgNPs in purified water, Ex & AgNPs: dispersed AgNPs in the Jaft extract, control: untreated cell line. Percentage of cytotoxicity is expressed relative to untreated controls (^*^significant  *P* < 0.05).

As observed from Figure 8(a) by increasing in concentration of AgNPs in Jaft extract cytotoxicity in MCF-7 cells was increased. The IC_50_ value of dispersed AgNPs in Jaft extract was 0.04 *μ*g/mL whereas this value for isolated and dispersed AgNPs in purified water was 50 *μ*g/mL. In agreement with previous reports [13, 15], AgNPs in Jaft extract and dispersed AgNPs in purified water have no significant cytotoxicity at their lower concentration on normal cell lines (Figure 8(b)). The results show that the cytotoxic effects of biosynthesized AgNPs increased in presence of Jaft extract on cancer cell line. This behavior can be attributed to the compounds in the extract that enhanced the activity of the AgNPs.

## 4. Conclusions

For the first time biosynthesis of AgNPs by using aqueous oak fruit hull (Jaft) extract was developed and optimized. TEM and DLS analyses have shown the biosynthesized AgNPs were spherical in shape with an average size of 40 nm. The results indicated cytotoxic activity of dispersed AgNPs in the Jaft extract was higher than AgNPs on MCF-7 breast cancer cell line. Therefore, the Jaft extract containing silver nanoparticles might be a potential alternative agent for human breast cancer therapy. The results of zeta potential show that the biosynthesized AgNPs have a long-term stability. Nanoparticles in extract solution were stable for one month.
